# A response to the Vancouver call for action: addressing the needs of early career scientists in radiation protection

**DOI:** 10.1007/s00411-025-01145-z

**Published:** 2025-09-17

**Authors:** Ämilie L. Degenhardt, Patrizia Kunert, Viktoria Herzner, Sehajpreet Gill, Nazanin Love, Jad Abuhamed, Giorgia Stendardo, Kim Lea Sennhenn, Warren A. John, Prabal Subedi

**Affiliations:** 1 Federal Office for Radiation Protection BfS, Ingolstädter Landstraße 1, 85764 Oberschleißheim, Germany; 2 Department for Technical Radiation Protection , Austrian Agency for Health and Food Safety, Spargelfeldstraße 191, 1220 Vienna, Austria; 3 Leibniz Institute for Prevention Research and Epidemiology - BIPS, Achterstraße 30, 28359 Bremen, Germany; 4 Research Group Environmental Economics, Centre of Environmental Sciences, Hasselt University, 3590 Diepenbeek, Belgium; 5 Health Sciences Unit, Tampere University, Arvo Ylpön katu 34, 33520 Tampere, Finland; 6 National Center for Innovative Technologies in Public Health, Italian National Institute of Health, Rome, Italy Viale Regina Elena 299, 00161; 7 GSI Helmholtzzentrum für Schwerionenforschung GmbH, Darmstadt, Germany Planckstraße 1, 64291

**Keywords:** Early career scientists, Radiation protection, Mentorship and networking, Vancouver call for action, RadoNorm, ECRad

## Abstract

Early career researchers, professionals, and scientists (ECRs) are essential to the future of radiation protection, a field that increasingly relies on interdisciplinary collaboration and innovation. In line with the principles outlined in the Vancouver Call for Action for Radiation Protection Researchers, an ICRP (International Commission on Radiological Protection) initiative, this article explores the current landscape for ECRs through the lens of survey data, initiative outcomes, and the establishment of the Early Career in Radiation Protection Network (ECRad). Drawing on a Europe-wide survey of 47 ECRs, the study identifies key areas of concern: while there is strong intrinsic motivation to remain in the field, perceived feasibility is often hampered by job insecurity, fragmented institutional support, and lack of structured mentorship. Although most respondents participate in existing networks such as ICRP, EURADOS, and IRPA, many reported unmet expectations, particularly in mentorship, sustained peer interaction, and accessible professional development. The formation of the RadoNorm Early Career Researcher Council (ECRC) responded directly to these gaps, demonstrating that self-organized, ECR-led initiatives can significantly enhance a sense of belonging and interdisciplinary engagement. However, structural barriers – such as time, funding, and short-term project support – persist, echoing the Vancouver Call for Action’s call for improved education, training, and retention. In conclusion, while considerable progress has been made in addressing the needs of ECRs in radiation protection, gaps that threaten the long-term vitality of the field remain. The findings affirm the urgency of coordinated action among institutions, networks, and funding bodies to invest in and empower the next generation of radiation protection professionals.

**Supplementary Information:**

The online version contains supplementary material available at 10.1007/s00411-025-01145-z.

## Introduction

At its 2022 Vancouver symposium, the International Commission on Radiological Protection (ICRP) issued a call to strengthen global expertise in radiation protection (Rühm et al. 2023). This initiative, referred to as the Vancouver Call for Action, highlighted the growing risk of declining competence in radiation protection disciplines. This loss of expertise threatens the safe use of radiation technologies and diminishes public trust. To ensure robust quality assurance and metrological traceability in radiation science, a sufficiently large and well-trained workforce is essential. Such capacity is critical for safely optimizing the use of ionizing radiation across a range of sectors, including medicine, material science, radioactive waste management and space exploration. Ultimately, this supports broader societal competence in understanding both the benefits and risks of ionizing radiation. In response to this challenge, the ICRP proposed several actions. First, governments and funding agencies need to recognize radiation science as a long-term societal priority. Second, research institutions need to launch and sustain long-term research programs. Third, universities need to establish undergraduate and graduate-level programs and promote job opportunities in this field, raising awareness among students and young professionals of the importance of radiation research. Fourth, clear and simple language should be used when communicating with the public and decision makers about radiological protection. And fifth, it is imperative to raise general awareness about the safe and appropriate use of radiation and radiological protection. This should be achieved by educating and training key individuals who in turn share and spread information to others.

The ICRP’s Call for Action received broad support from major organizations in medical physics, nuclear safety, radiation dosimetry, radiological protection, metrology and social sciences, as documented in a support letter (Mazzoni et al. 2024). The signatories stressed the need for well-educated scientists and professionals to ensure the continued relevance of radiation protection systems across diverse societal sectors. A similar call had already been made by the International Radiation Protection Association (IRPA) (Bryant et al. 2021). They identified that although there is an increased demand for radiation protection, in multidisciplinary fields, there is also a growing skill gap due to widespread retirement of senior personnel. To address this, IRPA recommended efforts to improve the public image of the profession, support early career development through mentoring and networking initiatives, and secure access to education and training opportunities.

Collectively, these calls underscore the need for structured and sustained support for early career researchers, professionals, and scientists (ECRs) in radiation protection. This need is already recognized by key radiation protection organizations through initiatives such as conference travel grants, online workshops, research exchange programs, and mentorship schemes. In 2022, the EU-funded RadoNorm project established a dedicated network for ECRs working in radiation protection, particularly in relation to radon and naturally occurring radioactive material (NORM). This network has been offering training courses and regular forums for Master students, PhD candidates, and postdoctoral researchers. However, since RadoNorm is a time-limited project, a group of ECRs has initiated efforts to continue and expand this network independently as the Early Career in Radiation Protection Network (ECRad).

In this paper, our response to the Vancouver Call for Action presents the perspectives of ECRs from diverse radiation protection disciplines. It includes an overview of existing support programs, a summary of past and ongoing initiatives to establish a new ECR network, results from a survey of opinions from 47 ECRs around the world, and a discussion of current challenges and unmet needs. Key questions are addressed: Is there a sustained need for a new network? Will it be accepted by the community? And how can it be implemented and financed on a long-term basis?

## The emergence of ECR networks in radiation protection

Europe benefits from a well-established infrastructure of key organizations and networks dedicated to radiation protection. Major international bodies such as the IRPA and the ICRP also offer valuable platforms and opportunities that can be utilized by ECRs. Additional opportunities for ECR involvement arise through national and international projects focused on specific topics in radiation protection, many of which allocate dedicated resources to support ECRs. An overview of current and recently concluded ECR programs and initiatives is provided in Table 1. Within this organizational landscape, IRPA serves as an umbrella organization for national radiation protection societies worldwide. These societies not only represent their respective countries within IRPA but also actively foster the growth of ECRs through a variety of educational and networking initiatives. In Europe, around 30 countries are represented within IRPA through 23 associate societies (Supplementary Information, SI1) (IRPA 2025). Many of these national societies were founded between the 1950 s and 1970 s, in response to the growing importance of radiation protection in medicine, industry, and research. Supporting ECRs remains a core mission for most of these societies.

**Table 1:** Current and recently concluded ECR programs and initiatives in radiation protection

Organization (name of ECR network)	Status	Offers for ECRs	Radiation protection field	Website (accessed on 17.06.2025)
ENEN2+	Ongoing	Travel grants, ENEN PhD event & prize, webinars, trainings	Nuclear	https://enen.eu/index.php/phd-events/
ERRS (NGenR²)	Ongoing	Young Investigator Award (YIA)	Diverse	https://www.errs.eu/en/next-generation-radiation-researchers
EURADOS	Ongoing	Grants, Young scientist award, EURADOS schools (winter schools), webinars, trainings	Dosimetry	https://eurados.sckcen.be/en
EURAMED	Ongoing	Prize for ECRs at European radiation protection week (ERPW), trainings in cooperation with other organizations	Medical	https://www.euramed.eu/
IAEA	Ongoing	Women in nuclear (WiN) initiative, Marie-Sklodowska-Curie Fellowship Programme, Lise-Meitner Programme, webinars, trainings	Nuclear	https://www.iaea.org/services/education-and-training/training-courses
ICRP	Ongoing	Cousins Award for Young Scientists and Professionals, Mentoring programme	All fields	https://www.icrp.org/index.asp
IRPA (IRPA YGN)	Ongoing	Travel grants, Montreal Fund supporting attendance of young professionals at IRPA Congresses, Young Career Professionals Award, contests (e.g. Movie Contest, Identity Card Contest), involvement in task groups	All fields	https://www.irpa.net https://www.irpa.net/ypn/index.asp
ISORED	Ongoing	Mentoring programme (MINI), involvement in working group	Epidemiology, Dosimetry	https://www.isored.org/
MELODI	Ongoing	Travel grants, prizes for dissertation or thesis, workshops	Low dose	https://melodi-online.eu/
NERIS	Ongoing	Trainings	Nuclear, Emergency	https://www.eu-neris.net/
PIANOFORTE	Ongoing	Travel grants, funding opportunities for ECR activities, trainings	All fields, Europe	https://pianoforte-partnership.eu/
RENEB	Ongoing	Travel grants, trainings	Dosimetry, Emergency	https://www.reneb.net/
SHARE	Ongoing	SHARE award at the annual RICOMET conference	Social sciences	https://www.ssh-share.eu/

Although there is a strong infrastructure for ECRs in radiation protection communities, several challenges still persist. Within individual countries, initiatives aimed at engaging ECRs exist but are frequently limited in duration, typically ceasing once project-specific funding ends. These initiatives offer a range of opportunities, including training workshops, networking events, mentoring programs, travel grants, scholarships, and thesis awards (Supplementary Information, SI1). However, there is limited systematic evaluation of their long-term effectiveness in supporting ECR development, and it remains unclear which types of programs are most sought after or most needed by ECRs. Consequently, insights into effective strategies rely largely on anecdotal evidence and the experiences of senior members and participating ECRs.

Another important challenge relates to the interdisciplinary nature of radiation protection itself. Effective practice in this field requires collaboration across diverse disciplines, including biology, medical physics, clinical medicine, epidemiology, social sciences, and communication. However, current ECR networks tend to remain within disciplinary “niches,” and opportunities for interdisciplinary networking are limited. Although key organizations have acknowledged the importance of supporting ECRs and the current lack of opportunities, the voices of ECRs themselves are often fragmented and not presented in a unified manner. In consideration of the similar regulatory and institutional frameworks within continents, an important step forward in addressing ECR’s challenges could be the establishment of a coordinated umbrella organization or network at the continental level that fosters interdisciplinary collaboration and strengthens ECR engagement across all areas of radiation research.

### The RadoNorm early career researcher Council (ECRC)

One example of the successful integration of ECRs into the scientific community is the RadoNorm project. Launched in 2020 under the Horizon 2020 program, RadoNorm aims to improve the management of risks associated with radon and NORM (Kulka et al. 2022). This €18 million project brings together 57 European institutions specializing in radiation protection research and risk management. Recognizing the critical challenges faced by the radiation protection field in recruiting, training, and retaining the next generation of professionals (Bryant et al. 2021), a substantial portion of the project’s budget is dedicated to education and training activities. This includes the funding of 25 PhD projects and several postdoctoral positions, all coordinated under a dedicated work package. In addition to supporting research positions, this work package provides travel grants to facilitate ECR participation in scientific conferences, training courses, and exchange visits. It also allocates resources to project partners for organizing specialized training courses focused on radon and NORM topics. Recently, initiatives in the project were expanded to include grants supporting open access publication for young researchers. The project’s comprehensive approach to nurturing ECRs in radiation research was one of the aspects specifically commended by the European Commission during its initial evaluation of the project proposal.

At the start of the project, the RadoNorm education and training work package (WP7) organized an Early Career Researcher Day, held online due to the COVID-19 pandemic, where ECRs presented their individual research projects (RadoNorm 2021). The event attracted strong participation and generated considerable enthusiasm, but the online format limited networking opportunities and did not sustain further momentum within the ECR community. To build on the initial enthusiasm, the project’s executive board facilitated an in-person gathering at the second annual meeting, aiming to strengthen ECR engagement. During this meeting, ECRs formed the RadoNorm Early Career Research Council (ECRC), elected a chairperson, scientific secretary, and work package representatives, and agreed to hold monthly online meetings to discuss research results, provide peer support, and organize events (John et al. 2022).

The monthly meetings showcased the ECRs’ work across various work packages and, given the multidisciplinary nature of the project, fostered constructive discussions and out-of-the-box thinking. Importantly, they provided a safe environment where ECRs could explain their work in simple terms without fear of judgment, encouraging open questions and knowledge-sharing across disciplinary boundaries, an element often lacking in formal academic settings, where a high level of prior knowledge is assumed. This supportive atmosphere not only built confidence but also fostered camaraderie among the ECRs, which had been lacking at the project’s start. Such camaraderie is well established among senior generations within the radiation protection community and has facilitated intense collaboration, particularly among European institutions. Establishing a similar close-knit network among the next generation was therefore seen as essential to ensuring an effective succession of researchers.

The RadoNorm travel grants were extensively used by ECRs to present their research at numerous conferences, thereby contributing significantly to the dissemination of the project’s results and facilitating their integration into the radiation protection community (RadoNorm 2025b). The RadoNorm research stay grants further supported longer exchange visits and research stays (RadoNorm 2025a). Based on personal testimonies, notable outcomes of these networking opportunities include helping ECRs secure new career positions within European radiation protection institutions, broadening their research horizons, and establishing contacts with internationally recognized organizations such as the ICRP and WHO.

#### Training courses organized by RadoNorm ECRC

The RadoNorm ECRC organized several training courses within the frame of WP7, aimed at educating an innovative and critical new generation of experts in radiation protection. Funding within WP7 enabled the RadoNorm ECRC to deliver high-quality training and strengthen the emerging network of ECRs in the field.

The first course, held in April 2023 at Stockholm University, brought together RadoNorm PhD students and postdoctoral researchers for a four-day event on transdisciplinary communication (Degenhardt 2023). Participants received training on presenting research across disciplines and to the public, with lectures from RadoNorm experts and invited speakers. The course, “Transdisciplinary Communication in Radon and NORM”, concluded with a visit to the Vasa Museum, highlighting the importance of effective communication with stakeholders for improving radon protection.

The second course “Career Management and Perspectives in Radon and NORM”, held in Prague in April 2024 and hosted by the Czech National Radiation Protection Institute (SURO), focused on career management and development (Degenhardt 2024). Sessions covered career paths in radiation protection, data visualization, time and stress management, social media use, and the role of artificial intelligence (AI) in research, and concluded with participant feedback and a tour of Prague.

The third course, organized in March 2025 in Granada, addressed the application of AI in scientific research with a focus on radiation protection (Sennhenn 2025). The program, “AI in Science: Key Knowledge, Applications and Challenges”, included expert lectures and hands-on sessions on generative AI, prompt engineering, data-centric methods, and ethical issues. Eleven participants attended, reporting high satisfaction with the training and networking opportunities. Interest in follow-up activities and future collaboration with the AI Granada Foundation was expressed.

#### Challenges faced by RadoNorm

While RadoNorm achieved important milestones, it also revealed key challenges and opportunities for future growth. Recruiting qualified PhD students and postdoctoral researchers proved difficult, with some positions requiring multiple rounds of applications to secure candidates. This shortage highlighted the fact that foundational education in radiation research needs strengthening at the university level, as emphasized in the Vancouver Call for Action (Rühm et al. 2023). It also reflected a shifting landscape, where careers in industry increasingly draw young talent away from academia. In several cases, promising PhD candidates left projects midway to pursue more attractive professional opportunities.

The RadoNorm ECRC faced its own challenges in maintaining engagement. Although RadoNorm management aimed to encourage ECRs to build and lead initiatives independently, only a fraction of the members fully embraced this opportunity. Motivating ECRs to take initiative and stay committed proved more complex than simply providing funding or opportunities. Some ECRs struggled with heavy project workloads, competing commitments, or lacked sustained interest.

A contributing factor was the late formal establishment of the council, towards the end of the second project year, meaning that the first RadoNorm ECRC-organized training course took place about two and a half years into the project. For many PhD candidates on three-year contracts, this coincided with the demanding final stages of their research, limiting their ability to participate in RadoNorm ECRC activities. Introducing career development opportunities earlier, such as the time and stress management sessions offered in the second training course in Prague, might have improved participation and retention. This experience underlines the importance of integrating soft-skills development and peer-networking early in large-scale research projects.

Finally, as the majority of RadoNorm ECRC members were PhD students, council membership naturally declined as students completed their projects and left. The few who remained sought ways to sustain the network beyond the end of the RadoNorm project.

### The emergence of the early career in radiation protection network (ECRad)

Upon further reflection, broader questions emerged:

- Were there other ECRs in radiation protection also seeking a community for mutual support and professional development?
- How would ECRs stay connected after the conclusion of the RadoNorm project?

In response, the idea emerged to establish a dedicated network, the Early Career in Radiation Protection Network (ECRad), with the goal of expanding the professional horizons of ECRs by offering opportunities to connect with peers and experienced professionals from diverse backgrounds. ECRad could also provide practical advice on career development, mentorship opportunities, and introductions to the various radiation protection networks active across Europe.

A meeting was organized to assess whether there was a genuine need for a new network, considering that many platforms, projects, and associations in Europe already offer similar support within the field of radiation protection.

#### Meeting in Munich

The first ECRad meeting, funded by European Partnership for Radiation Protection Research (PIANOFORTE), was jointly organized by the German Federal Office for Radiation Protection (BfS), Tampere University (TUNI), University of Hasselt and the Belgian Nuclear Research Centre (SCK CEN) on 13–14 May, 2024, at the Helmholtz Forschungszentrum Campus in Munich (PIANOFORTE 2024).

An in-person meeting was organized to facilitate meaningful interactions, knowledge exchange, and professional relationship-building among participants. The aims of the event were to identify the specific needs of ECRs in radiation protection and to establish an ECR network connecting groups within European projects, associations, and platforms.

It brought together both senior and junior speakers and representatives from a broad range of organizations, platforms, partnerships, associations, and committees, including: UNSCEAR, ICRP, BfS, SCK CEN, PIANOFORTE, IRPA, RadoNorm, the German Commission on Radiological Protection (SSK), the European Radiation Dosimetry Group (EURADOS), the Multidisciplinary European Low Dose Initiative (MELODI), the International Society of Radiation Epidemiology and Dosimetry (ISoRED), Social Sciences and Humanities in Ionising Radiation Research (SHARE), the Austrian Association for Radiation Protection (ÖVS), Munich Young Radiation Researchers (MYRR), and the German-Swiss Association for Radiation Protection (FS).

A central theme of the workshop was the need for sustained, long-term support for ECRs, whether through training, recognition of achievements, and professional development opportunities within their organizations. Discussions focused on the importance of structured mentoring programs, access to funding, and the challenges commonly faced by ECRs. The value of both formal mentoring and informal networks, such as those facilitated by social media, was emphasized as essential for building connections and supporting emerging scientists.

Participants and speakers recommended the establishment of a centralized platform, which will act as a comprehensive hub for ECRs, providing information about funding opportunities, research programs, and career development resources. Expanding financial support through travel grants, research and secondment scholarships was highlighted as key to enabling ECRs to attend conferences, workshops, and other events critical to their career progression. The low attendance of ECRs at networking events was a recurring issue, leading to the unanimous agreement on the need for more attractive and relevant participation opportunities, including flexible event formats and enhanced financial support.

A valuable discussion also emerged regarding the use of inclusive language. It was recognized that terminology can inadvertently exclude individuals, especially in international contexts where meanings may vary. To address this, the term “scientist” was proposed in its broadest sense, encompassing researchers and professionals in radiation protection regardless of academic background or job title. In this paper, the acronym ECR is used to refer inclusively to researchers, professionals, and scientists working in the field.

#### Meeting in Rome

One of the main outcomes of ECRad’s first meeting in Munich was the decision to establish an annual in-person meeting of the network at the European Radiation Protection Week (ERPW) in November 2024 in Rome. With financial support from PIANOFORTE and logistical assistance from the local organizers, the Italian National Institute of Health (ISS) and the Italian Association of Radiation Protection (AIRP), a hybrid ECRad meeting was organized as an official side event of the ERPW. A social event organized at a local restaurant fostered informal networking opportunities among ECRs.

Attendees included ECRs based in Italy, Belgium, Germany, Austria, England, Japan, and Australia. Because the meeting was integrated into the ERPW conference, representatives of various radiation protection organizations and research platforms were able to attend and present their programs dedicated to ECRs in person. For example, IRPA, EURADOS, EURAMED, and local networks from Germany and Italy contributed to the sessions, and representatives of RadoNorm and PIANOFORTE joined a panel discussion on guiding the next generation.

An important part of the program focused on the ECRs themselves, giving them the opportunity to share their experiences with support programs, such as travel grants, offered by radiation protection organizations. During a live survey, the personal needs of attendees regarding a new network were discussed interactively. It became evident that while many participants are already members of existing networks, there is still a demand for a new umbrella network that connects these different initiatives.

Participants supported the idea of holding annual ECRad meetings within the context of ERPW. They felt this would motivate other ECRs to attend and contribute, given ERPW’s status as one of the most significant annual events in the radiation protection community. The survey was modified and opened up online to more participants after the conference.

## Survey on networking needs for early career scientists

To better understand the specific needs and challenges faced by ECRs in radiation protection, a survey (Supplementary Information SI2) was conducted among ECRs participating in various initiatives. The survey aimed to identify key factors influencing career development, including mentorship, funding opportunities, interdisciplinary collaborations, and professional training. It also sought to determine whether ECRs see a need for a new network and whether they would be interested in actively participating in such an initiative. Data collection was conducted via an online platform, with the survey open from 24 February 2025 to 31 March 2025. The main objectives were to assess the primary barriers ECRs encounter in networking and career development, to determine the most valuable resources and opportunities for ECRs in radiation protection, and to gather insights on preferred formats for networking events and mentorship programs.

### Participant profile

A total of 47 ECRs completed the survey. The majority, 68.1%, were affiliated with research or academic institutions, while others were involved in regulatory or authority bodies (14.9%), medical or clinical settings (12.8%), or industry (4.3%). Regarding age distribution, 40.4% of respondents were between 30 and 35 years old, 31.9% were between 25 and 30, and 27.7% were over 35. In terms of experience, 31.9% were pursuing a PhD, 25.5% identified as junior postdoctoral researchers, and 19.1% held more senior academic positions such as principal investigator or professor. Additionally, 12.8% worked as industry professionals, 6.4% selected the category “other,” and 4.3% were completing a Master’s degree.

### Survey results

**Fig. 1:**
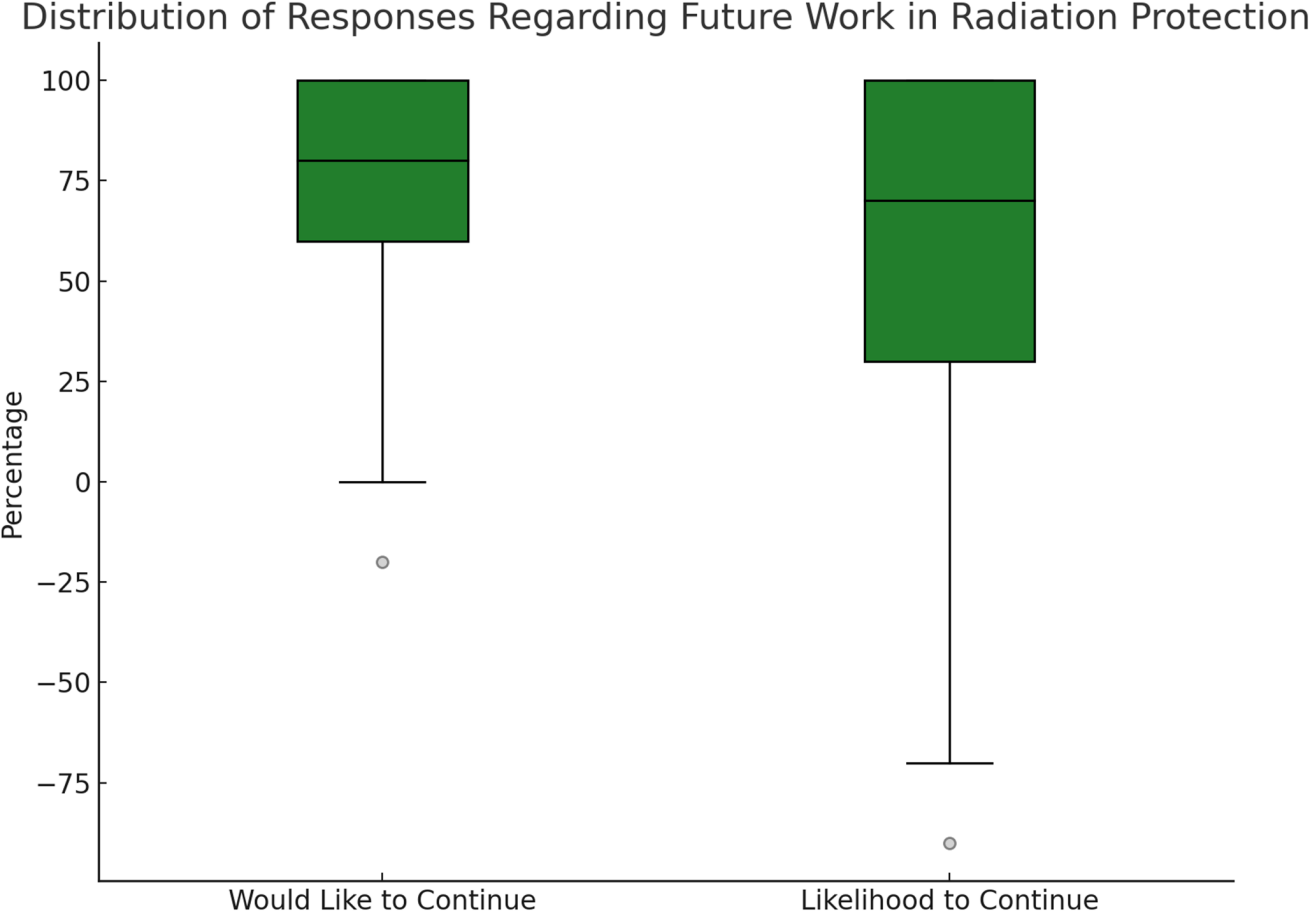
Distribution of responses regarding the desire of the ECRs to work in radiation protection and practical likelihood to continue working in the field. An anonymous online survey was conducted and 47 participants took part in the survey. The boxes represent the interquartile range (25th–75th percentile), with the horizontal line indicating the median. Whiskers extend to 1.5 times the interquartile range, and outliers are shown as individual points

Attitudes towards the future of radiation protection as a career were mixed. While some participants held a positive view, describing the field as “promising,” “stable,” or “secure”, others expressed negative sentiments, often citing poor job prospects, funding uncertainty, and low visibility. To better distinguish between motivation and feasibility, respondents were asked two related but separate questions: whether they *wanted* to continue working in radiation protection, and whether they *expected* to do so given their current circumstances (Fig. 1). The responses reveal a consistent gap between these two dimensions. While 38.3% gave the maximum score (100) for their desire to stay in the field, only 31.9% did so for their likelihood of continuing. Desire ratings were strongly clustered at the positive end of the scale, whereas likelihood ratings were more widely dispersed. This pattern suggests that although many respondents are highly motivated, their expectations are tempered by structural constraints such as job scarcity, contract instability, and limited funding opportunities.

A paired t-test and Wilcoxon signed-rank test confirmed the significance of this gap (*p* = 0.0088 and *p* = 0.0080, respectively). Free text responses reinforced this interpretation, frequently referencing job scarcity, contract instability, and inadequate funding as barriers to remaining in the field. This gap between desire and likelihood was especially visible among PhD candidates, who, despite showing willingness to remain in radiation protection, reported notably lower average expectations of doing so. Their responses also displayed the greatest variability, underscoring the uncertainty that characterizes this early career stage and pointing to the impact of limited permanent opportunities and broader systemic barriers.

**Fig. 2:**
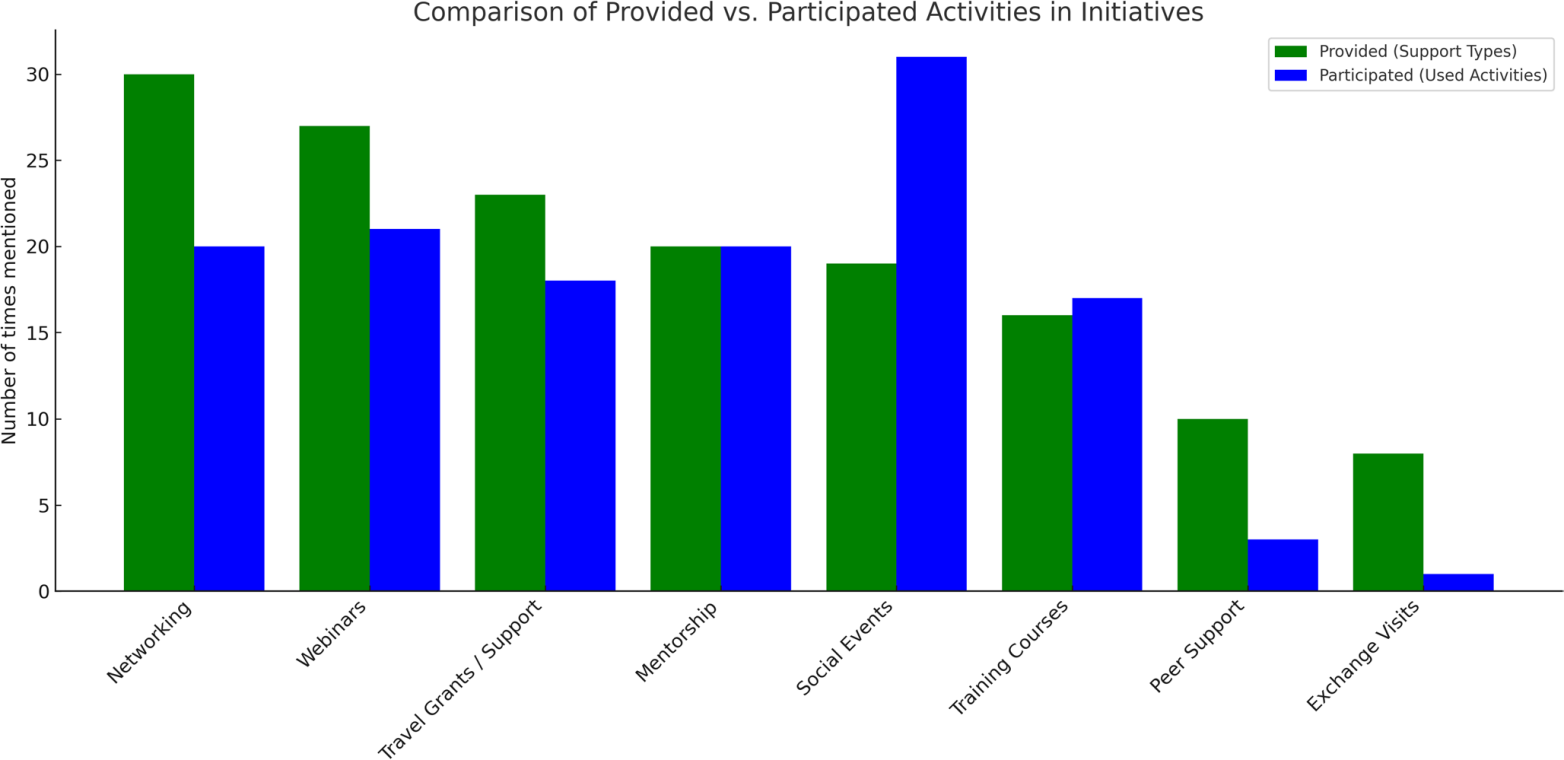
Services offered by and used in early career initiatives. An anonymous online survey was conducted and 47 participants took part in the survey. Green bars denote the activities provided by the initiatives ECRs are enrolled in and blue bars denote the activities that the ECRs have actually used

Most respondents (83.0%) reported membership in a radiation protection network, most frequently citing international organizations and platforms such as the ICRP, EURADOS, IRPA, and ALLIANCE. Among these, 76.6% indicated their networks offered initiatives specifically targeting ECRs. These initiatives commonly took the form of mentorship programs (e.g., the ICRP Mentorship Program), involvement in task groups, and early career events, and offered a range of opportunities including networking, webinars, training courses, social events, and financial support such as travel grants and awards. Notably, networking, social events, mentorship, and webinars were, by a considerable degree, the most frequently used services across the networks (Fig. 2).

However, while this variety of offerings points to a broad and supportive landscape, the findings also reveal that some personal needs and expectations remain unmet. The most frequently cited issue was related to mentorship. Several responses referred to the limited availability of mentors, with remarks suggesting a need for more structured and sustained guidance, particularly in the form of detailed and constructive feedback. In addition to mentorship, comments pointed to the absence of formal communication platforms that could facilitate continuous peer engagement beyond annual events or conferences. While social events provided opportunities to meet other ECRs, collaborative formats such as joint projects or shared scientific activities were reported as largely unavailable. Financial support, particularly for attending international meetings, was also mentioned as a gap in several responses, especially given that conferences are often necessary for meaningful networking.

Furthermore, some respondents expressed a desire for centralized platforms that could provide updates on relevant opportunities, including travel, training, and open positions. A few noted that their networks met their expectations or that they were still in the early stages of involvement and unable to assess. While most feedback focused on practical aspects of support, one response raised concerns about structural barriers that inhibit equitable participation. Overall, while many networks offer valuable forms of support, the findings highlight the importance of consistent, inclusive, and interactive structures that respond to the evolving needs of ECRs across different institutional and geographic contexts.

Statistical analysis (Chi-square tests) revealed no significant association between respondents’ age or level of experience and their membership in radiation protection networks (*p* > 0.05), suggesting that network participation was not shaped by these demographic variables. However, this finding should be interpreted with caution given the small sample size and the associated limitations in statistical power. The possibility of self-selection bias must also be considered, as the survey may have primarily reached individuals already engaged with or interested in networking opportunities.

When asked whether a new network for ECRs is needed, 36.2% answered affirmatively, while a majority (55.3%) selected “Not sure,” reflecting uncertainty about its added value. Only 8.5% believed no new network was necessary. Age and level of experience did not significantly influence these responses. The high proportion of uncertain responses suggests a need for clearer articulation of how a new initiative would differ from existing efforts and address unmet needs. Expectations were shown for a Europe-wide network centered on improving networking opportunities, particularly those that foster genuine connection through research exchanges, joint activities, and social events.

Many requested mentorship programs, hands-on training, and expert Q&A sessions, pointing to a strong desire for structured professional development. Additionally, several respondents highlighted the importance of interdisciplinary inclusion and international collaboration, suggesting that the network should extend beyond Europe to include global peers. Some respondents also called for better promotion and visibility of the network itself, ensuring accessibility and engagement across diverse backgrounds and regions. Importantly, these expectations align with the perceived shortcomings of current networks, reinforcing the case for a complementary rather than duplicative platform.

Importantly, the results highlight that despite the diverse hindrances to attractively retain ECRs, the great majority have a willingness to stay. A key recommendation to the current networks and European radiation research platforms would be to place an emphasis on mentoring opportunities, not simply that of a PhD student and supervisor, but also mentoring opportunities with other senior scientists to transfer knowledge and experience apart from the academic sphere. In this light, the ICRP Mentorship Programme and ISoRED MINI program are already paving the way (ICRP 2025; ISoRED 2025).

Networks are also recommended to continue to provide opportunities for ECRs to meet and engage in networking and social events. Moreover, carrying out periodic assessments of the needs and desires of ECRs would be prudent as they are subject to change over generations.

## Lessons learned and challenges

### Lessons learned

Forming a new network requires engaged peers who are willing to organize regular online meetings or workshops, manage online outreach and advertising, secure funding for network activities, and foster a culture of active participation among other ECRs. This is a substantial undertaking, especially given that ECRs are already managing significant workloads and often working under time-limited contracts within their PhD or postdoctoral programs. As a result, continual recruitment of new members is essential to sustain the network over time. In addition, support from employers is crucial in enabling ECRs to contribute actively to such initiatives. This can be achieved by providing dedicated funding opportunities that recognise and value engagement in community-building efforts.

Nevertheless, a self-organized ECR network offers valuable opportunities for interdisciplinary collaboration among the next generation of radiation protection scientists. Such networks can foster a stronger sense of belonging to the scientific community, recognize the contributions of individual ECRs, and serve as a motivation for them to remain in the field beyond their initial projects. Importantly, active involvement of ECRs in shaping their professional environment also sends a strong message to stakeholders, international organizations, and universities, encouraging them to continue and intensify their support for future generations.

The ECRad survey revealed that most respondents expressed a clear desire to remain in the field. However, external factors, such as contract instability and insufficient funding, often leave ECRs discouraged. There is a clear need to offer more stable career opportunities, moving beyond the model of funding short-term PhD projects, toward investing in long-term training and providing pathways to sustainable professional roles in radiation protection.

Mentoring emerged as a particularly valued initiative, with programs from organizations such as the ICRP and ISORED being well utilized by ECRs. Survey respondents indicated a strong desire for additional mentoring opportunities. For instance, while the RadoNorm project did not offer mentoring beyond the traditional PhD supervisor-student relationship, this was identified as a missed opportunity given the project’s scale and diversity of partners. Such initiatives should be more actively developed, including within the PIANOFORTE framework.

Mentoring can bridge the gap between senior experts and newcomers, transmitting critical professional and life skills that accelerate career development. To make the field more attractive to new entrants, it is crucial that senior professionals recognize the importance of transgenerational engagement and take an active and humble role in mentoring. Effective mentoring is a demanding and sustained commitment, involving not just providing answers but building relationships that encompass teaching, guidance, sponsorship, professional socialization, and ongoing moral support (Ragins and Kram 2007).

Moreover, peer mentoring, where support occurs horizontally among colleagues at similar career stages, is gaining traction and offers unique benefits not always achieved through traditional hierarchical models (Bussey-Jones et al. 2006). In light of these findings, it would be valuable to conduct a dedicated study exploring best practices in mentoring within radiation protection, and to develop a formal framework for such programs, as has been established in other scientific fields (Davis 2013).

### Challenges

ECRs require the active support of their mentors and supervisors to participate meaningfully in professional networks and organizations alongside their regular workloads. Supervisors should motivate and facilitate their active contribution. Such involvement can help ECRs develop a sense of community and see tangible benefits for their professional growth. Building a sense of belonging is challenging; therefore, while online meetings are valuable, in-person meetings are strongly recommended to foster deeper connections. The availability of dedicated funding, such as that currently provided within the PIANOFORTE partnership, is essential to enable these activities.

Conference organizers should consider ways to increase the inclusion of ECRs, for example by offering opportunities to organize dedicated sessions and by providing discounted attendance fees to help lower financial barriers. Strengthening the ECR community within radiation protection is likely to enhance motivation and retention, provided that there are sufficient permanent positions and clear career pathways available.

The three-stage IRPA model for building competence in radiation protection encompasses: (1) attracting future professionals, (2) developing knowledge and skills in radiation protection, and (3) supporting retention, further development, and career progression (Bryant et al. 2021). One major challenge identified in this model is communicating the attractiveness of the profession to those outside the field. Meaningfulness at work is a key driver of job satisfaction and plays a crucial role in attracting and retaining new recruits (Oh and Roh 2019). While meaningfulness is multifactorial, emphasizing the corporate social responsibility inherent in radiation protection, such as improving public health, advancing environmental protection, and responding to societal needs, can make the field more appealing. Personal testimonies from established professionals, as well as clear evidence of policymaker engagement and investment, are also important for inspiring new entrants. It should be made clear that radiation protection is a critical concern in routine contexts: safeguard of cancer patients and health workers in hospitals, protection of children in schools from radon exposure, and proactive preparation for future emergency scenarios.

A key challenge for emerging networks such as ECRad is ensuring sustainability beyond the duration of major projects like RadoNorm. When such projects end, the dedicated funding, structural support, and time allocated for early-career development are typically withdrawn, threatening the network’s momentum and continued opportunities for ECRs. To ensure long-term success, alternative support mechanisms and ongoing commitment from the broader scientific community are essential.

The experience of the RadoNorm project illustrates both the successes and the challenges involved in building a sustainable and engaged network for ECRs in radiation protection. While the project offered valuable opportunities for training, collaboration, and career development, it also brought to light several structural, cultural, and motivational factors that can limit the long-term effectiveness of such networks. The challenges outlined below, drawn from the RadoNorm experience as well as similar initiatives, highlight the complexities associated with fostering lasting ECR engagement in this field.

1. *Fragmentation of the Field and Networking Gaps*: Radiation protection is an interdisciplinary field, incorporating natural sciences, medicine, engineering, social sciences, and policy. However, most existing networks are national, discipline- or institution-specific, limiting exchange and collaboration. This lack of connectivity across diverse niches creates barriers for ECRs who seek to understand the big picture of the field and prevents the organic development of broader professional communities.
2. *Sustainability and the Funding Trap*: Many ECR activities, including those under large-scale projects like RadoNorm, are dependent on fixed-term project funding. Once the project ends, so do the initiatives regardless of their impact or popularity. The initial energy and community formed often dissipate without institutional mechanisms or follow-up funding to support continued engagement.
3. *Recruitment and Retention Difficulties*: Despite the availability of funding, several RadoNorm positions required multiple recruitment cycles, and some remained unfilled. This highlights both a shortage of trained personnel in foundational aspects of radiation protection and a broader trend of declining interest in academic careers among younger generations. Additionally, career paths outside academia, particularly in industry, are often perceived as more attractive, leading to high turnover and reduced continuity within networks.
4. *Limited and Uneven Engagement*: While the establishment of the RadoNorm ECRC was initially met with enthusiasm, sustained participation proved challenging. Competing priorities, such as demanding research workloads, external commitments, or a general lack of interest, led to waning involvement over time. This demonstrates that even well-funded and structured initiatives cannot rely solely on resources; they also require consistent motivation, relevance, and perceived value from participants.
5. *Representational Gaps and Missing ECR Voices*: Despite being acknowledged by senior bodies as critical to the future of the field, ECRs still often lack a unified platform to express their needs and shape research and policy agendas. In many cases, their voices are filtered through senior representatives rather than being directly incorporated into governance and decision-making. The idea of starting ECRad was, in part, a response to this representational gap, aiming to unify and amplify ECR perspectives across institutions and initiatives.
6. *Need for In-Person Networking and Community Building*: While digital events and platforms offer accessibility and convenience, they cannot fully replicate the depth and quality of in-person interactions. RadoNorm’s initial online ECR Day highlighted this limitation, as it failed to generate sustained engagement. In contrast, in-person events such as the Munich ECRad meeting proved more effective in fostering meaningful professional connections and long-term collaboration.
7. *Structural Turnover and Leadership Transition*: ECR-led initiatives face a natural challenge of turnover as members complete their studies or move into new roles. The RadoNorm ECRC experienced diminishing numbers as PhD students graduated and moved on, leaving gaps in leadership and continuity. Without a mechanism for succession planning such networks are at risk of dissolving over time.

While the dedication and enthusiasm of ECRs are invaluable assets in building strong networks, these challenges cannot be overcome by their motivation alone. The complexities and barriers identified require structural changes and long-term support from the broader radiation protection community. Effective solutions must come from a collective effort, involving not only the ECRs themselves but also the established networks, institutions, and policymakers. The success of future ECR networks will depend on creating a framework that ensures continuity, inclusivity, and the integration of diverse disciplines within the field.

## Conclusion and future directions

The field of radiation protection offers promising career opportunities for early-career researchers, professionals, and scientists (ECRs), but also presents significant challenges. The survey and analysis indicate that while many ECRs are motivated and eager to remain in the field, issues such as job insecurity, limited funding, and fragmented networking opportunities can make this difficult.

Current networks provide useful resources such as mentorship programs, webinars, social events, and financial support. However, there are still gaps, especially in consistent mentorship, ongoing communication platforms, interdisciplinary collaboration, and funding for attending international meetings. Many ECRs expressed interest in a centralized, Europe-wide network that could offer more coordinated, inclusive, and interactive support tailored to their needs.

Mentorship plays a key role in helping ECRs grow professionally and feel connected. This requires senior scientists to actively support and guide younger colleagues beyond just academic supervision. Peer mentoring also shows promise and could be further developed. A strong sense of community also needs to be built through both online and in-person events.

Sustaining these networks over time is a challenge, especially since many rely on short-term project funding and face natural turnover as members move on. For networks to thrive, they need ongoing institutional support, clear leadership succession plans, and integration with broader European and global initiatives. Learning from existing frameworks like MELODI and PIANOFORTE can help maintain momentum and make the best use of available resources.

In summary, addressing the challenges faced by ECRs requires effort from everyone involved: researchers, mentors, institutions, policymakers, and funders. The next generation of scientists can be supported by stable career paths, improved mentorships, and inclusive and interdisciplinary networks.

The ECRad initiative has made significant strides toward understanding and addressing the unique needs of ECRs in radiation protection. The events in Munich and Rome, supported by PIANOFORTE and various international partners, demonstrated the power of dedicated, in-person networking opportunities to foster a sense of community, enhance visibility, and encourage active participation among ECRs across Europe and beyond.

A key insight from this initiative is that while motivation among ECRs remains high, structural support is essential for the translation of that motivation into sustainable engagement and long-term retention. Mentoring programs, like those pioneered by ICRP and ISORED, must be expanded and institutionalized. Moreover, ECRs need to be actively involved in shaping the networks they are part of, with senior professionals committing time and effort to foster meaningful mentoring relationships. Physical events remain crucial for building trust and community, and sustained funding will be required to ensure their continuation.

## Supplementary Information

Below is the link to the electronic supplementary material.

Supplementary Material 1.

## Data Availability

The results of the ECRad Survey 2025 can be found here: [10.20348/STOREDB/1213].
