# Supplementary material for: A response to the Vancouver call for action: addressing the needs of early career scientists in radiation protection
Source: Radiat Environ Biophys. 2025 Sep 17;64(4):581–93. doi: 10.1007/s00411-025-01145-z (PMC12700991; doi:10.1007/s00411-025-01145-z)
Supplement: Supplementary file 1 — Supplementary Material 1. [file 411_2025_1145_MOESM1_ESM.docx]

**A Response to the Vancouver Call for Action: Addressing the Needs of Early Career Scientists in Radiation Protection**

Ämilie L. Degenhardt, Patrizia Kunert, Viktoria Herzner, Sehajpreet Gill, Nazanin Love, Jad Abuhamed, Stendardo Giorgia, Kim Sennhenn, Warren A. John, and Prabal Subedi

**Supplementary Information**

SI1: Associate societies of the International Radiation Protection Association (IRPA) offering support for ECRs.

| **Society (Country)** | **Status** | **Offers for ECRs** | **Website (accessed on 17.06.2025)** |
| --- | --- | --- | --- |
| ÖVS (Austria) | Ongoing | Travel and research grants, prizes for dissertation or thesis, webinars, mentoring program, job portal, excursions | <https://strahlenschutzverband.at/> |
| BVS-ABR (Belgium) | Ongoing | Young scientist award, science contest, trainings, job portal | <https://bvsabr.be/> |
| CRPA (Croatia) | Ongoing | Young scientist award, symposium with poster award and oral presentation award, lectures, trainings | <https://www.hdzz.hr/en> |
| ČSOZ (Czech Republic) | Ongoing | Prizes for significant contribution to a scientific paper or a university diploma thesis | <https://csoz.suro.cz> |
| NSFS (Denmark, Finland, Iceland, Norway, Sweden) | Ongoing | Young scientist award | <https://nsfs.org> |
| FS (Germany,Switzerland) | Ongoing | Travel grants, prizes for dissertation or thesis, webinars, mentoring program, job portal, excursions | <https://www.fs-ev.org/home> |
| AIRP (Italy) | **Coming Soon** | Reduced society congress fee, young award & thesis award contests (monetary prizes, free congress participation, one-year subscription to Health Physics and one-year membership for finalists and winners), scientific schools, trainings | <https://www.airp-asso.it> |
| SPPCR (Portugal) | Ongoing | Reduced society congress fee | <https://www.sppcr.pt> |
| SEPR (Spain) | Ongoing | Young scientist award, lectures about career opportunities, job portal | <https://www.sepr.es> |
| SRP (United Kingdom) | Ongoing | Young professional award, webinar, trainings, mentoring program, excursions | <https://srp-uk.org> |

Note: Societies listed are those for which networking offers to ECRs were confirmed via direct response or publicly available information. Societies that did not respond to our request are not included; however, these societies may also offer relevant opportunities for ECRs that are not reflected here.

SI2: ECRad Survey 2025 – Questionnaire

Platform used: [www.tedme.com](http://www.tedme.com)

Duration of survey: 24.2.2025 – 31.3.2025

| **General questions** | |
| --- | --- |
| **Question 1** | **What is your line of work?** |
| Mandatory answer | Yes |
| Question type | Single choice |
| Answer choices | Research / Academia  Industry  Medical / Clinical  Authority / Regulator  Other |
| Follow-up question 1 trigger | When “Other” is selected |
| Follow-up question 1 | **Please specify.** |
| Follow-up question 1 mandatory answer | No |
| Follow-up question 1 question type | Free-text |

| **Question 2** | **How old are you?** |
| --- | --- |
| Mandatory answer | Yes |
| Question type | Single choice |
| Answer choices | Under 20 years old  20 – 25 years old  25 – 30 years old  30 – 35 years old  Over 35 years old |
| Follow-up question | N/A |

| **Question 3** | **What is your level of experience?** |
| --- | --- |
| Prompt | Please select your current occupational level. |
| Mandatory answer | Yes |
| Question type | Single choice |
| Answer choices | Doing my Bachelors  Doing my Masters  Pursuing a PhD  Junior postdoc  Senior researcher / principal investigator  Industry professional  Other |
| Follow-up question 1 trigger | When “Other” is selected |
| Follow-up question | **Please specify.** |
| Follow-up question 1 mandatory answer | No |
| Follow-up question 1 question type | Free-text |

| **Initiatives in radiation protection field** | |
| --- | --- |
| **Question 4** | **Are you part of any radiation protection network(s)?** |
| Prompt | E.g. EURADOS, ICRP, IRPA, MELODI, ALLIANCE, etc. |
| Mandatory answer | Yes |
| Question type | Single choice |
| Answer choices | Yes  No |
| Follow-up question 1 trigger | When “Yes” is selected |
| Follow-up question 1 | **Please specify which one(s).** |
| Follow-up question 1 mandatory answer | Yes |
| Follow-up question 1 question type | Free-text |

| **Question 5** | **Do(es) the network(s) you are part of have an initiative for early career scientists / professionals?** |
| --- | --- |
| Prompt | None |
| Mandatory answer | Yes |
| Question type | Single choice |
| Answer choices | Yes  No |
| Follow-up question 1 trigger | When “Yes” is selected |
| Follow-up question 1 | **Please specify which initiative(s).** |
| Follow-up question 1 mandatory answer | Yes |
| Follow-up question 1 question type | Free-text |

| **Question 6** | **What does this initiative provide?** |
| --- | --- |
| Prompt | Please select as many as possible. |
| Mandatory answer | Yes |
| Question type | Multiple choice |
| Answer choices | mentorship  networking  training courses  webinars  peer support  social events  travel grants / support  exchange visits  none  other |
| Follow-up question 1 trigger | When “other” is selected |
| Follow-up question 1 | **Please specify.** |
| Follow-up question 1 mandatory answer | Yes |
| Follow-up question 1 question type | Free-text |

| **Question 7** | **If you have made use of / participated in any of these activities, please say which.** |
| --- | --- |
| Prompt | Please select as many as applicable. |
| Mandatory answer | Yes |
| Question type | Multiple choice |
| Answer choices | mentorship  networking  training courses  webinars  peer support  social events  travel grants / support  exchange visits  none  other |
| Follow-up question 1 trigger | When “other” is selected |
| Follow-up question 1 | **Please specify.** |
| Follow-up question 1 mandatory answer | Yes |
| Follow-up question 1 question type | Free-text |

| **Question 8** | **What does your network lack in terms of your personal needs / expectations?** |
| --- | --- |
| Prompt | None |
| Mandatory answer | No |
| Question type | Free-text |

| ***Future of radiation protection*** | |
| --- | --- |
| **Question 9** | **How do you feel about the future of radiation protection as a career?** |
| Prompt | None |
| Mandatory answer | Yes |
| Question type | Free-text |

| **Question 10** | **Regardless of circumstances, would you like to continue working in radiation protection?** |
| --- | --- |
| Prompt | None |
| Mandatory answer | No |
| Question type | Tendency |
| Answer option | Choosing on a scale from -100 to +100  -100: *Not at all*; +100: *Very much* |

| **Question 11** | **How likely are you to continue working in radiation protection?** |
| --- | --- |
| Prompt | None |
| Mandatory answer | Yes |
| Question type | Tendency |
| Answer option | Choosing on a scale from -100 to +100  -100: *Not at all*; +100: *Very much* |
| Follow-up question 1 trigger | If selected value is less than 10 |
| Follow-up question 1 | **What is hindering you from staying in the field of radiation protection?** |
| Follow-up question 1 question type | Multiple choice |
| Follow-up question 1 mandatory answer | Yes |
| Follow-up question 2 trigger | If “other” is selected in follow-up question 1. |
| Follow-up question 2 | **Please specify if possible.** |
| Follow-up question 2 mandatory answer | No |
| Follow-up question 2 question type | Free-text |
| Follow-up question 1 trigger | If selected value is more than 20 |
| Follow-up question 3 | **What incentives motivate you to stay in the field?** |
| Follow-up question 3 question type | Multiple choice |
| Follow-up question 3 mandatory answer | Yes |
| Follow-up question 4 mandatory answer | No |
| Follow-up question 4 trigger | If “other” is selected in follow-up question 3. |
| Follow-up question 4 | **Please specify.** |
| Follow-up question 4 mandatory answer | No |
| Follow-up question 4 question type | Free-text |

| ***New Network*** | |
| --- | --- |
| **Question 12** | **Do you think a new network is needed for early career scientists / professionals?** |
| Prompt | Please consider ECRad as a new network. |
| Mandatory answer | Yes |
| Question type | Single choice |
| Answer choices | Yes  No  Not sure |
| Follow-up question 1 trigger | If “Yes” is selected in follow-up question 3. |
| Follow-up question 1 mandatory answer | No |
| Follow-up question 1 | **Why do you think so?** |
| Follow-up question 1 question type | Free-text |
| Follow-up question 2 trigger | If “No” is selected in follow-up question 3. |
| Follow-up question 2 mandatory answer | No |
| Follow-up question 2 question type | Free-text |

| **Question 13** | **What do you expect from a new Europe-wide radiation protection network for early career scientists / professionals?** |
| --- | --- |
| Prompt | None |
| Mandatory answer | No |
| Question type | Free-text |

| **Question 14** | **If a new network such as ECRad were to be formed, how likely are you to be actively involved?** |
| --- | --- |
| Prompt | None |
| Mandatory answer | Yes |
| Question type | Tendency |
| Answer option | Choosing on a scale from -100 to +100  -100: *Very unlikely*; +100: *Very likely* |
| Follow-up question 1 trigger | If selected value is less than 40 |
| Follow-up question 1 mandatory answer | No |
| Follow-up question 1 | **Please say what hinders you from being involved.** |
| Follow-up question 1 question type | Free-text |
